# Effects of Mild Fatigue on Biomechanics of Single Leg Landing in Young Male Volleyball Players

**DOI:** 10.3390/s24216811

**Published:** 2024-10-23

**Authors:** Taisen Li, Leonid Vladimirovich Kapilevich, Junru Chen

**Affiliations:** 1 Department of Sport Tourism, Sport Physiology and Medicine, National Research Tomsk State University, 634050 Tomsk, Russia; litais@stud.tsu.ru; 2 Central Research Laboratory, Siberian State Medical University, 634050 Tomsk, Russia; 3 Faculty of Sport and Health Sciences, University of Jyväskylä, 40014 Jyväskylä, Finland; junru.h.chen@jyu.fi

**Keywords:** volleyball, landing, young players, fatigue

## Abstract

Objective: To investigate the effects of mild fatigue on the biomechanics of the lower limbs of young male volleyball players while performing single leg landing tasks. Methods: A total of ten young male volleyball players were recruited as participants in this study. After the single leg landing was performed, we compared the performance between those with and without fatigue (post- and non-fatigue, respectively). Kinematics and kinetics were collected using instruments, and related captured data were imported into OpenSim to analyze the hip, knee, and ankle joints. Results: We found that the ankle dorsiflexion angle at initial contact was significantly decreased in the post-fatigue group compared to the non-fatigue one. Meanwhile, the peak gluteus maximus force, peak gluteus medius force, peak tibialis posterior force, and peak gastrocnemius force significantly increased. There were no significant differences in the hip and knee joint flexion angles as well as the quadriceps and biceps femoris long head forces between the two groups. Conclusions: Mild fatigue can affect the performance of single leg landing, and players need to control the lower limbs by generating a higher muscle force to cope with the instability induced by fatigue. In a fatigued state, following initial contact with the ground, a decreased ankle dorsiflexion angle necessitates an increase in gastrocnemius and tibialis posterior muscle force to maintain stance during landing.

## 1. Introduction

Volleyball is one of the top five international sports, being played in 220 countries and by 800 million players worldwide [1]. It is characterized by the short duration and high intensity that occurs successively during a training session or a match, which puts high demands on the musculoskeletal system [2]. Consequently, volleyball players are at risk of musculoskeletal injuries [3,4]. In a study of injuries in 486 volleyball players, Verhagen and colleagues found that the players had an average of 1.8 lower limb injuries per 1000 h [5]. A survey of the 2013–2015 NCCA volleyball tournament showed lower limb injuries happened with an average of 2.03 per 1000 h. Jumping, spiking, and landing are the primary risk factors leading to injuries in volleyball [6]. Nevertheless, landings are a typical occurrence in volleyball and landing is a major cause of lower limb injuries, especially when performing single leg landing. Marquez et al. showed that elite male volleyball players tend more to practice the landing habits in single leg landing than double leg landing [7]. The existing studies have paid less attention to mild fatigue in volleyball games and training, instead focusing more on a kind of fatigue that tends to exhaustion. However, such intense fatigue does not often occur in games and training. At the same time, there is not enough evidence and discussion to analyze the relationship between mild fatigue and volleyball injuries in the existing studies.

Fatigue is a crucial factor in single leg landing, which increases the risks of injuries [8,9]. A common method to determine fatigue is to monitor the attenuation rate of the continuous jump height, according to which fatigue can be classified into medium fatigue (attenuation of 80%) [8,10], severe fatigue (attenuation of 70%) [11,12], and mild fatigue (attenuation of 90%) [13]. A previous study by Wnorowski et al. found that volleyball players’ jumps did not attenuate below 90% of their maximum height due to fatigue until the end of the game [14]. However, there are few studies on the mild fatigue protocol. Hence, choosing a mild fatigue protocol with continuous jumps to 90% of the maximum jump height appears valuable.

Compared to adults, studies show that young male players were more prone to injury due to their immature musculoskeletal development [15,16]. Lower limb injuries were also found to coincide with the presence of muscle fatigue in young players [17]. A study of injuries among young male volleyball players in Japan showed that lower limb injuries were the most common both in training and competition conditions, accounting for 4.24 per 1000 h [18].

As several of the aforementioned epidemiological research studies have demonstrated, there is a need to focus on the level of fitness of this group. However, the changes in the landing biomechanics of young volleyball players in fatigue remain poorly understood due to the lack of sufficient relevant studies.

The purpose of this study was to investigate whether mild fatigue could lead to changes in the biomechanics of single leg landings in an experimental environment. Our hypothesis was that mild fatigue results in changes in the kinematics, kinetics, and muscle force of single leg landings in people with fatigue compared to those without it.

## 2. Materials and Methods

### 2.1. Participants

The participants of this study were recruited at the Dalian Sports School. Ten U14–U16 Junior male volleyball players (age: 15.2 ± 0.7 years; height: 184.9 ± 4.7 cm; mass: 72.9 ± 7.1 kg; training age: 3.1 ± 0.2 years) were enrolled in this study. All participants had right-leg dominance. They had no musculoskeletal injuries of the lower limbs in the previous six months and did not engage in strenuous exercises within 24 h prior to the experiment. Participants had practiced professional exercises (including volleyball exercises, jumping exercises, and other physical exercises) at least five times a week for at least two years. We recruited a total of 11 individuals, one of whom was excluded from the study due to a violation of the above criteria. They were informed about the potential risks of injury and signed an informed consent form. The study followed the guidelines of the Declaration of Helsinki (Table 1).

### 2.2. Experiment Procedure

**Figure 1:**
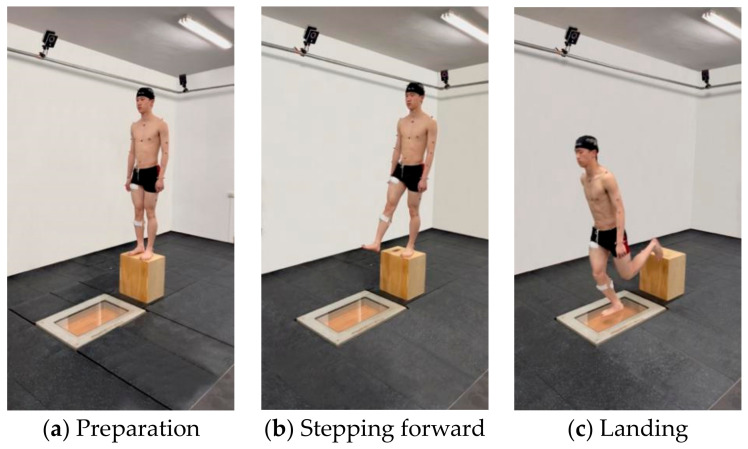
Task steps: (**a**) Stand with both feet on the box and look ahead. (**b**) Naturally extend the dominant leg forward. (**c**) Land on the force platform with the dominant foot.

During the experiment, participants were topless, barefoot, and wearing tight shorts. A five-minute warm-up was performed before the formal test. The warm-up session was conducted in accordance with the routine previously employed by the participants prior to their daily volleyball training sessions. This routine included dynamic stretching of various body parts and jogging. Before the warm-up, the instructions on how to complete the single leg landing task were explained and displayed to them. In each trial, the participants were asked to perform three single leg landings from a 45 cm box as naturally as possible with a toe–heel touchdown and then leave the force platform (Figure 1). A successful trial was recognized if they landed on the force platform using the dominant leg and walked out of the force platform completely.

### 2.3. Fatigue Protocol

We asked the participants to accomplish a maximum touch height experiment, and the highest of the three jumps represented the jumping ability. After smoothly completing three non-fatigue single leg landing trials, the participants were instructed to continuously jump on both legs in place until the touch height was decreased to less than 90% of the maximum touch height. At this time, they were considered to have reached a state of mild fatigue.

### 2.4. Data Collection

**Figure 2:**
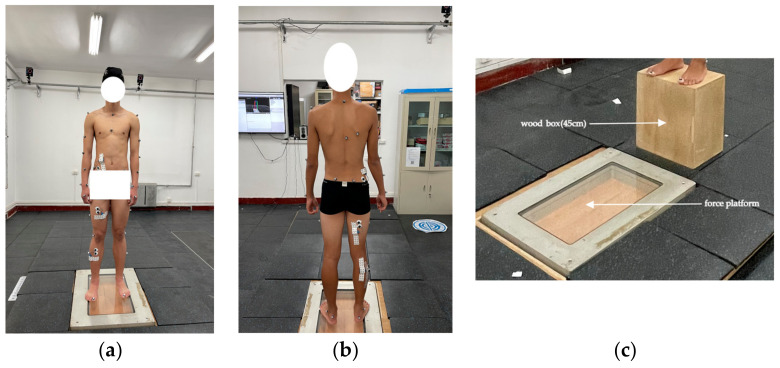
(**a**,**b**): Retro-reflective markers attached to the participant’s skin. (**c**): Test facility setup.

Kinematics were recorded using a Vicon Nexus system with 8 cameras (Oxford Metrics, Oxford, UK) sampling at 100 Hz, and the ground reaction force (GRF) was captured using an AMTI force platform (Advanced Mechanical Technology, Watertown, MA, USA) sampling at 1000 Hz. A total of 39 reflective markers were placed on each participant’s skin surface, including on the head × 4, arm × 12, acromion × 2, clavicle × 1, sternal raphe × 1, 10th thoracic vertebrae × 1, 7th cervical vertebrae × 1, subscapularis × 1, anterior superior iliac spine × 2, posterior superior iliac spine × 2, mid-thigh bone × 2, lateral condyle of the femur × 2, mid-calf bone × 2, lateral malleolus × 2, heel × 2, and first metatarsal bone × 2. Finally, a box with a height of 45 cm was placed 50 cm from the center of the force platform (Figure 2).

### 2.5. Data Processing

The landing phase in this study was defined as the time interval from the initial foot contact to the maximum knee flexion [12]. The vertical GRF and the joint moments were normalized to the body mass (Nm/kg).

**Figure 3:**
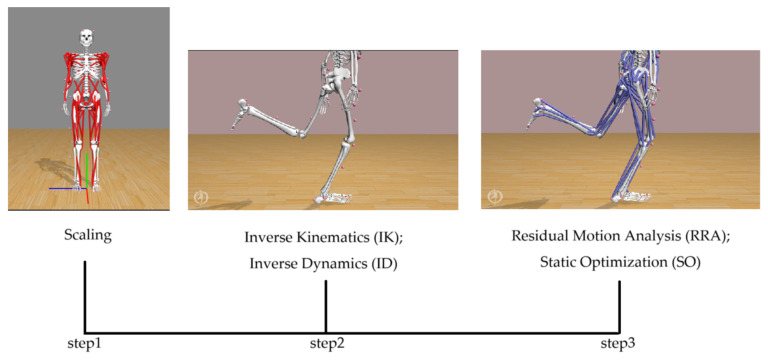
Workflow of OpenSim.

In order to simulate the single leg landing maneuver, the “Gait 2354_Simbody” model (https://simtk.org/projects/ulb_project) in OpenSim (4.2, Simtk.org) was accessed on 7 May 2022. Participants were given head circumference, chest circumference, bilateral arm length, palm width, waist circumference, bilateral thigh length, knee width, bilateral calf length, and bilateral foot length measurements before the start of the experiment for use in personalized model building during the simulation (Table 2). The procedure of scale was adapted to match the anthropometric parameter of each subject. A series of joint angles were obtained by applying inverse kinematics (IK), and moments in the lower limb were obtained by running the inverse dynamics (ID). The inconsistencies between the measured kinematics and the GRFs were minimized using the residual reduction algorithm (RRA), such that a modified body model was realized and static optimization (SO) was utilized based on the RRA to calculate the muscle force (Figure 3). The muscle forces were normalized to the bodyweight (BW).

### 2.6. Workflow of OpenSim

Input Data and Model Setup

1. Skeletal model: Typically based on medical imaging (e.g., CT, MRI) or standardized human skeletal models.

Motion capture data: Data from a motion capture system (e.g., C3D format) using markers placed on the human body to measure joint motion.

External force data: External forces are collected using the force platform. Muscle model: Includes muscle paths, musculoskeletal geometry, and physiological parameters (e.g., optimal fiber length, tendon length).

2. Inverse kinematics (IK)

Objective: To estimate the skeletal poses and joint angles based on known motion capture data.

Mathematical principle: Minimize the difference between the actual position of the motion capture markers and the corresponding points in the model. This is typically solved using optimization methods by minimizing the objective function:

$$\min\sum_{i=1}^{n}w_{i}(obs_{i}-{\mathrm{model}}_{i}{)}^{2}$$

where $obs_{i}$ is the observed position of the i-th marker, ${model}_{i}$ is the position of the corresponding model marker, and $w_{i}$ is the weight coefficient.

3. Inverse dynamics (ID)

Objective: To compute the joint moments and forces required to produce the observed motion, based on the known kinematic data (e.g., joint angles) and external forces.

Mathematical principle: Based on the Newton–Euler equations, applying Newton’s second law F = ma and the conservation of angular momentum, the joint torques and reaction forces are computed from the known joint kinematics and external forces:

$$\tau =J^{T}\cdot f-m\cdot \dot{\upsilon }$$

where τ represents the joint torques, *J* is the Jacobian matrix, *f* is the external forces, *m* is the mass, and $\dot{\upsilon }$ is the acceleration.

Joint angle calculation

Mathematical principle: The joint angles are derived from inverse kinematics and are typically represented as Euler angles or quaternions, depending on the type of joint (e.g., ball-and-socket, hinge joints).

4. Residual reduction algorithm (RRA)

Objective: To reduce residual forces by adjusting the model kinematics and dynamics, leading to more accurate simulation results.

Principle: the RRA adjusts the model’s mass distribution and kinematic data to better match the computed dynamic results with the measured data (e.g., external forces). It iteratively optimizes kinematic data and mass parameters by minimizing residual forces.

5. Static optimization (SO)

Objective: To compute individual muscle forces based on known joint moments.

Principle: Static optimization uses linear programming methods to minimize a cost function (usually the sum of the muscle activation levels or muscle forces) while satisfying the joint moment constraints. The cost function typically takes the following form:

$$\min\sum_{i=1}^{n}a_{i}^{2}$$

where $a_{i}$ is the activation level of the *i*-th muscle.

The goal is to find the optimal combination of muscle activations such that the joint moment equation is satisfied:

$$M=\sum_{i=1}^{n}R_{i}\cdot F_{i}$$

where M is the joint moment, $R_{i}$ is the moment arm of the muscle, and $F_{i}$ is the muscle force.

### 2.7. Statistical Analysis

All kinematics, kinetics and muscle forces were normalized to 100% of the landing phase, as shown in Figure 2 and Figure 3. The paired sample t-test was performed to examine the effect of fatigue on the landing biomechanics. The significance level was set at *p* = 0.05.

## 3. Results

**Figure 4:**
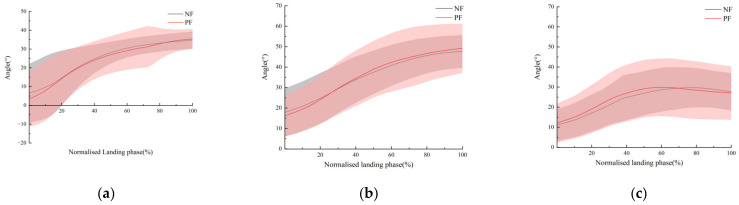
(**a**): ankle, (**b**): knee, (**c**): hip. These are the comparisons of the ankle, knee, and hip angles for single leg landing in non-fatigue (NF) and post-fatigue (PF) states, respectively.

Regarding the effect of mild fatigue on the kinematics, we found the post-fatigue group’s ankle dorsiflexion angles at initial contact (IC) were significantly different (*p* = 0.037) (Table 3) to the non-fatigue group. That means the dorsiflexion of the ankle was reduced. However, there were no significant differences in the variable kinematics in the hip, knee, and ankle angles. Angle variation trends of the ankle, knee, and hip joints in the sagittal plane are depicted in (Figure 4).

For the kinetics, we found significant changes in the peak plantarflexion ankle moment (*p* = 0.023). Meanwhile, no significant changes were observed in the GRF and sagittal of other joints (Table 4).

**Figure 5:**
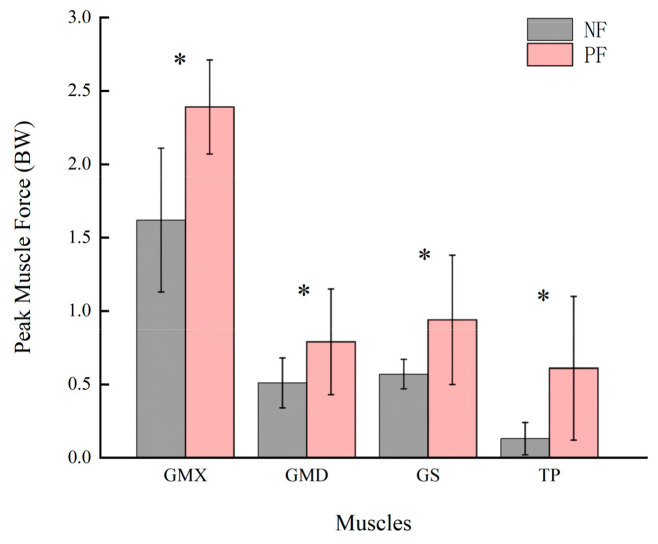
The comparison of the GMX, GMD, GS and TP’s peak muscle force for single leg landing in non-fatigue (NF) and post-fatigue (PF) states, respectively. * Significant differences with *p* < 0.05.

**Figure 6:**
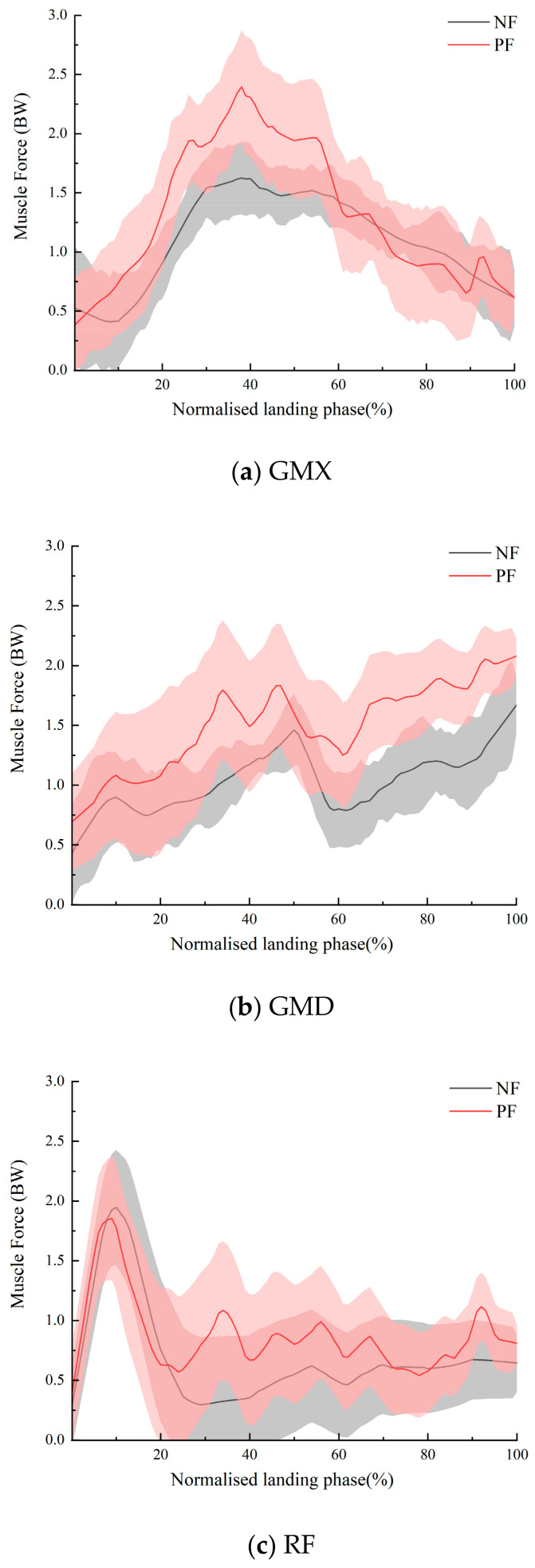

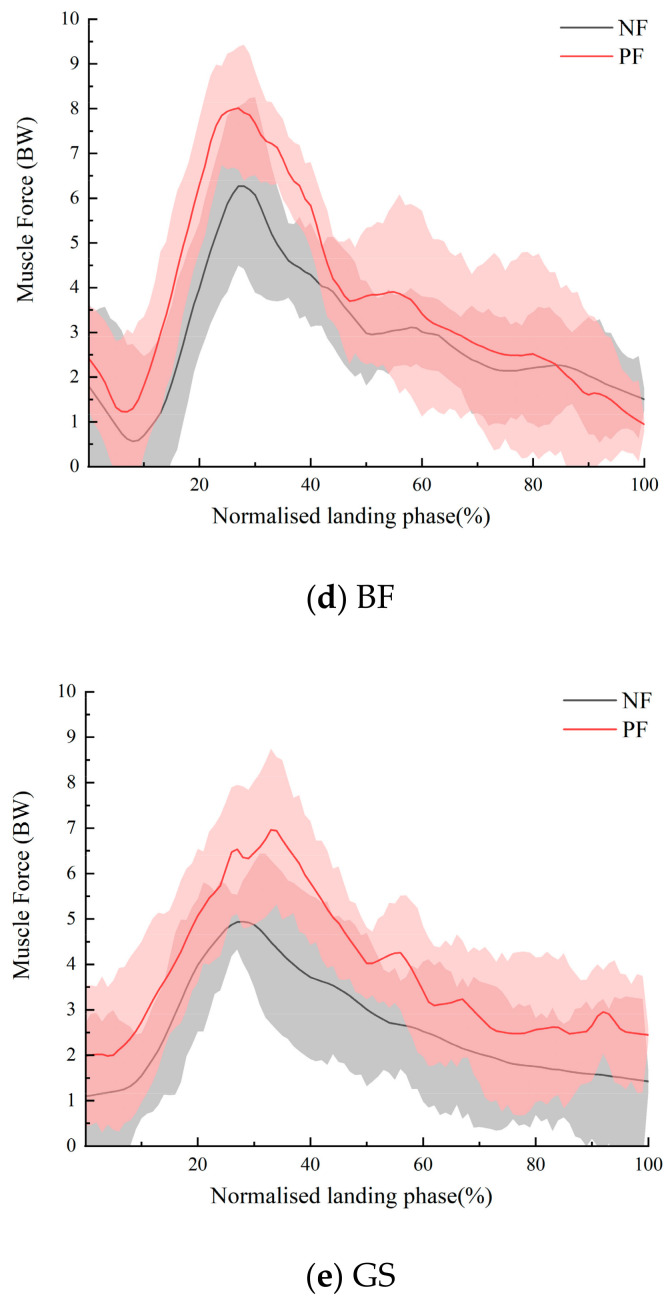
Calculated with a total of five muscle forces, comparing the NF to the PF of them.

With respect to the muscle force, we found significant differences in the peak gluteus maximus force (*p* = 0.009), peak gluteus medius force (*p* = 0.045), peak tibialis posterior force (*p* = 0.022), and peak gastrocnemius force (*p* = 0.036) due to fatigue, while no significant changes were observed among the other muscles (Table 5). The differences in peak muscle strength before and after fatigue for gluteus maximus, gluteus medius, tibialis posterior, peak gastrocnemius are illustrated in a bar graph (Figure 5). Furthermore, no notable discrepancies were identified in the calculated trend plots of muscle strength for the four muscles, with the exception of a slight augmentation in peak muscle strength following fatigue (Figure 6).

## 4. Discussion

The purpose of this study was to investigate the effects of mild fatigue on single leg landings performed by young male volleyball players. We used a fatigue protocol of a continuous jumping stimulation to achieve mild fatigue and collected data to compare the participants in two different states, which means the non-fatigue and post-fatigue cases. We found that the ankle dorsiflexion angle at IC was significantly smaller, while the knee joint flexion was significantly greater. Additionally, in terms of the muscle force, the gluteus maximus, gluteus medius, gastrocnemius, and tibialis posterior showed significantly greater peak forces. As a result, the findings partially support our hypothesis that some kinematics, kinetics variables, and muscle forces were changed due to mild fatigue.

The decrease in the ankle dorsiflexion angle suggests that mild fatigue impacts the lower limb biomechanics in young male volleyball players. This finding is intriguing because few studies have investigated the effects of mild fatigue on single leg landings. Indeed, there is a paucity of discussion of ankle changes in a state of mild fatigue. This is particularly the case in the context of volleyball. Nevertheless, the study conducted by Lin, H.T indicated that lower back fatigue may be a contributing factor to a stiff landing stance following jump shots and countermovement jumps in basketball [19]. This was evidenced by a notable reduction in the hip and knee flexion angles following fatigue. However, it is possible that this result may be related to the specific fatigue protocol employed for the lower back, which could still support the conclusions of the present study. A meta-analysis on the ankle and knee in landings indicated that the kinematic changes in the knee can be predicted by deep learning using the ankle dorsiflexion angle at initial landing [20]. This suggests that our experimental results and conclusions are in agreement with the aforementioned study’s conclusion that the ankle angle at initial landing is crucial for the knee. However, a key distinction is that our study observed the changes in the lower extremities before and after fatigue. These changes were found to be associated with the subject’s chosen landing strategy. Cortes et al. investigated landings after fatigue but used a more erect landing strategy [21]. This could be associated with the participants population, previous studies illustrated that the biomechanical variables of lower limbs were not always positively correlated with medium and severe fatigue and other mild fatigue protocols [12,22,23,24,25]. Our study enriches the domain to some extent. Specifically, mild fatigue did not increase the ankle dorsiflexion angle.

Instead, the participants chose a kind of non-conservative strategy to land. In other words, the participants adopted a more rigid landing strategy in order to bear the load from landing. Generally, as the degree of fatigue increases, the larger GRF is absorbed by the increase in the range of joint motion, which was considered a conservative protective mechanism. However, the participants in the current study did not actively control the range of motion of the joints of the lower limbs in order to increase it. An attempt was made to analyze this phenomenon from a muscular perspective. During the landing phase, the functions of the GS and TP muscles were responsible for plantarflexion and avoiding moving the tibia forward [26]. This study showed that the peaks of the GS and TP in a mild fatigue condition were significantly higher than those in a non-fatigue condition. In order to keep the body balanced, the participants had to raise the muscle force of the GS and TP to avoid excessive dorsiflexion of the ankle joint and flexion of the knee joint during single leg landings. Agonist and antagonist co-activation plays an important role in stabilizing the knee joint, especially after fatigue [27]. Nevertheless, in our study, the ankle joint was observed to be in passive dorsiflexion at the commencement of the landing phase. However, due to the passive nature of the movement, the muscle force of the tibialis anterior (TA), which is the agonist muscle, did not exhibit a significant increase during passive dorsiflexion, which may be one of the reasons why the ankle dorsiflexion angle did not increase but decreased. Unlike the muscle fatigue caused by medium or severe fatigue, mild fatigue does not cause a decrease in the muscle force, but it rather increases it, thus controlling the movements [28]. However, this might not necessarily appear to be a positive change, because increasing the hip–knee–ankle flexion angle during landing can prevent the bones and ACL from bearing more of the impact when absorbing the GRF, reducing the risk of injury [29,30].

It has been reported that players engaged in repeated taking off and landing with limited dorsiflexion would be unable to absorb the impact force and may also suffer from ACL rupture [31], Achilles tendon rupture [32], patella tendon rupture [33,34], ankle sprain [35], and chronic knee pain [36]. Hahn D’s study found the joint moment of multi-joint cooperative buckling to be smaller than that of single-joint buckling, so it is better to have a multi-joint cooperative absorption of the impact force [37]. However, in this study, the participants might have chosen a wrong strategy. Overall, the knee and hip joints’ angles in the sagittal plane did not significantly change at IC, which suggests that the lower limbs landed in a stiffer way due to mild fatigue. But, it seems that we have found a reason why the participants chose to land in this way.

Based on the experimental results, it was found that the participants’ GMD and GMX had significantly greater peak muscle strengths during mild fatigue, and the GMD and GMX could control hip abduction movements. At the same time, several studies have shown that the full activation or targeted training of the GMD and GMX can significantly improve postural control, such as in single leg balancing and single leg landing [38,39]. Such targeted training is most commonly seen in rehabilitation programs for ACL patients and in warm-up exercises for athletes [40,41]. In the present study, our participants demonstrated direct control of the hip movement and indirect control of the knee angle by increasing the peak muscle strength in the GMD and GMX, which may explain why the participants preferred a less conservative, rigid landing strategy for the landing task. Perhaps in future studies, we could have participants perform the landing task under different fatigue regimes and fatigue levels and observe and analyze their landing strategies. This fatigue protocol was chosen to more closely resemble the athletic characteristics of volleyball, with the aim of approximating as closely as possible a volleyball match environment with multiple jumps in a short period of time [42]. This is because, according to some studies, match-induced fatigue affects the knee position sense.

Match-induced fatigue can affect the knee joint position sense [43]. Several other central changes occur due to fatigue, including in proprioception and postural control [44]. Generally, the fatigue protocol can be divided into long-term and short-term protocols, depending on the conditions frequently encountered by the study participants. While the long-term fatigue protocol mainly involves running or cycling on a treadmill for long periods of time [12], the short-term fatigue protocol mainly involves squatting on single leg, continuously repeating vertical jumps and short sprints [45], and maximum repetitions on a leg-press machine [46,47]. However, since different fatigue protocols were used, many of the results are still controversial. It has been reported that increasing the flexion angle and motion range of the hip and knee during fatigue is the main strategy to decrease the GRF [12,25]. However, the results of Smeets and colleagues did not support this view, showing no significant kinematic differences even when using fatigue schemes similar to the former.

This study has some limitations in terms of the lower limb biomechanics, because the amount of participants what we recruit was not too much to prove a stronger basis for evidence. As well as the fact that we could not put the participants into a state of mild fatigue in time for a real game or training exercise, we were unable to gather them into the laboratory in time for the test equipment. And we only considered changes in the sagittal plane and did not include the kinematics and dynamics of the coronal plane. In future studies, the sagittal, coronal, and horizontal planes could be combined for analysis. This may offer a more complete picture of the biomechanical changes in the lower limbs. The results of this study may contribute to the analysis of the causes of lower limb injuries among young male volleyball players. For instance, building on our research using a finite element analysis of the differences in joint forces before and after fatigue will allow the field to have a clearer understanding of the lower limb joints during fatigue and to look for ways to prevent injuries due to landing on the ground while fatigued. 

## 5. Conclusions

This study demonstrated that mild fatigue resulted in a significant reduction in the ankle dorsiflexion angles in the sagittal plane and a significant increase in the peak muscle forces in the gluteus maximus, gluteus medius, gastrocnemius, and tibialis posterior muscles during single leg landings. The altered kinematics of the lower limb compelled the muscle groups responsible for controlling the landing stance to assume the loads borne by the lower limb joints from the ground through the exertion of enhanced muscle forces. Collectively, these changes in the biomechanical metrics indicated that the participants adopted a distinct landing strategy after mild fatigue compared to their non-fatigued state.

## Figures and Tables

**Table 1:** Participant inclusion and exclusion criteria.

Criterion	Inclusion Criteria	Exclusion Criteria
Age	13–18 years old	Younger than 13 or older than 18
Sex	Male	Female
Sport Experience	At least 2 years of volleyball training experience, currently on a school or club team	Lack of volleyball training experience or insufficient training volume
Health Status	No significant medical history, no lower extremity injuries, no recent surgeries	History of heart disease, hypertension, diabetes, or other chronic diseases; history of lower extremity injuries; recent surgeries
Training Volume	At least 3 training sessions per week, each session lasting at least 1 h	Insufficient training volume
Skill Level	Possesses good basic volleyball skills, especially landing technique	Poor technical skills
Strength Level	Passes strength tests to ensure adequate lower extremity muscle strength	Insufficient lower extremity muscle strength
Flexibility	Passes flexibility tests to ensure adequate range of motion in lower extremity joints	Insufficient flexibility
Dominant Hand and Foot	Dominant hand and foot are on the right side	Dominant hand and foot are on the left side
Other	Willingness to participate in the study and signed informed consent	Does not meet the specific requirements of the study

**Table 2:** This table presents the demographic and anthropometric data of the participants, which were collected prior to the experiment. The parameters include basic demographic information such as age, height, weight, and training experience, alongside key anthropometric measures essential for personalized model building in the OpenSim simulation. These measures include head circumference, chest circumference, bilateral arm length, palm width, waist circumference, bilateral thigh length, knee width, bilateral calf length, and bilateral foot length.

Participant’s Number	Age (years)	Height (cm)	Weight (kg)	Head Circumference (cm)	Chest Circumference (cm)	Arm Length (cm)	Palm Width (cm)	Waist Circumference (cm)	Thigh Length (cm)	Knee Width (cm)	Calf Length (cm)	Foot Length (cm)
1	15	181.2	65.9	56.4	91.8	58.4	8	72.4	44.2	10.1	37.7	24.7
2	16	179.0	81.4	59.3	88.7	58.6	8.1	66.4	52.5	10.3	32.9	25.9
3	16	187.5	79.7	56.4	88.5	54.2	8.3	67.7	55.2	10.2	36.8	25.1
4	15	183.8	65.2	56.3	87.9	57.7	8.8	72.2	51.5	10.6	39.7	23.4
5	15	189.2	71.9	60.7	95.4	61.4	8.1	72.3	54	10.7	38.4	23
6	15	182.1	67.4	56.8	98.1	62.8	8.7	70.6	48	11.1	36.5	23.7
7	15	190.3	75.8	55	89.8	59.4	8.1	63.4	50.6	9.8	36.4	25.1
8	16	186,7	69.6	57	92.3	58.9	8.8	72.2	48	11.3	38.1	23.2
9	16	185.5	76.4	56.5	91.4	61.9	8.3	71	46.6	9.2	37.8	25.8
10	14	188.0	68.7	58.4	88	66.2	8.3	69	49	11.1	39.1	22.2

**Table 3:** Effects of fatigue on the kinematics of the hip, knee, and ankle during single leg landing in mean (standard deviation [SD]).

	Non-Fatigue			Post-Fatigue		
	Hip	Knee	Ankle	Hip	Knee	Ankle
Joint angle at IC (°)	11.0 (7.9)	−17.7 (11.7)	6.3 (15.6)	11.9 (9.7)	−16.1 (10.2)	3.31 (4.7) *
Peak joint angle (°)	31.2 (19.9)	−47.6 (8.1)	34.8 (4.4)	32.8 (23.4)	−49.1 (11.9)	35.5 (5.4)
Range of motion (°)	20.5 (13.6)	29.8 (10.0)	28.6 (16.2)	21.9 (11.6)	35.2 (10.9) *	32.9 (15.7)

* Significant differences with *p* < 0.05.

**Table 4:** Effects of fatigue on the kinetics of hip, knee and ankle during single leg landing in mean (SD).

		Non-Fatigue			Post-Fatigue	
	Hip	Knee	Ankle	Hip	Knee	Ankle
Joint moment at IC (Nm/kg)	−1.7 (2.3)	−1.3 (1.8)	−0.4 (0.8)	−1.6 (2.0)	−1.5 (1.6)	−0.4 (0.9)
Peak joint moment (Nm/kg)	−4.7 (2.2)	2.8 (0.9)	−3.2 (0.3)	−5.3 (2.8)	2.3 (0.5)	−3.4 (0.8)

**Table 5:** The effect of fatigue on the muscle at IC and peak muscle force during single leg landing in mean (SD). GMX: gluteus maximus, GMD: gluteus medius, VI: vastus intermedius, RF: rectus femoris, BF: biceps femoris long head, GS: gastrocnemius, TP: tibialis posterior, TA: tibialis anterior.

	Non-Fatigue	Post-Fatigue
Muscle force at IC (BW)		
GMX	0.05 (0.03)	0.07 (0.11)
GMD	0.15 (0.15)	0.15 (0.19)
VI	0.09 (0.01)	0.20 (0.31)
RF	0.02 (0.01)	0.03 (0.04)
BF	0.18 (0.17)	0.23 (0.27)
GS	0.11 (0.04)	0.18 (0.19)
TP	0.07 (0.01)	0.17 (0.29)
TA	0.02 (0.01)	0.05 (0.07)
Peak muscle force (BW)		
GMX	1.62 (0.49)	2.39 (0.32) *
GMD	0.51 (0.17)	0.79 (0.36) *
VI	5.49 (3.76)	3.85 (3.75)
RF	0.27 (0.12)	0.39 (0.11)
BF	1.01 (0.58)	1.33 (0.72)
GS	0.57 (0.10)	0.94 (0.44) *
TP	0.13 (0.11)	0.61 (0.49) *
TA	0.03 (0.01)	0.41 (0.36)

* Significant differences with *p* < 0.05.

## Data Availability

The data presented in this study are available on request from the corresponding author.
